# Urinary incontinence in women 55 years and older: A scoping review to understand prevalence, incidence, and mortality of urinary incontinence during secondary care admission

**DOI:** 10.1177/17455057231179061

**Published:** 2023-06-16

**Authors:** Isobel McMillan, Lyndsay Hill, Robyn McCarthy, Ruth Haas-Eckersley, Margaret Russell, Julie Wood, Liz Doxford-Hook, Yu Fu, Linda McGowan, Heather Iles-Smith

**Affiliations:** 1 School of Health and Society, The University of Salford, Salford, UK; 2 Salford Royal NHS Foundation Trust, Salford, UK; 3 Leeds Teaching Hospitals NHS Trust, Leeds, UK; 4 Newcastle University, Newcastle upon Tyne, UK; 5 The University of Leeds, Leeds, UK

**Keywords:** hospital admission, incontinence of urine, mortality, older women, prevalence, scoping review

## Abstract

**Background:**

Up to 40% of older women living in the community experience urinary incontinence. In community settings, urinary incontinence impacts the quality of life, morbidity, and mortality rates. However, little is known about urinary incontinence and its impact on older women admitted to hospitals.

**Objectives:**

This scoping review aims to establish the current knowledge of urinary incontinence during hospital admission for women (⩾ 55 years of age) with three key objectives: (a) What is the prevalence/incidence of urinary incontinence? (b) What health conditions are associated with urinary incontinence? (c) Is there an association between urinary incontinence and mortality?

**Eligibility criteria:**

Empirical studies were included in assessing the incidence/prevalence of urinary incontinence during hospital admissions and its related morbidities and mortality rates. Studies which only included men or younger women (< 55 years of age) were excluded. Only articles written in English and conducted between 2015 and 2021 were included.

**Sources of evidence:**

A search strategy was developed, and CINAHL, MEDLINE, and Cochrane databases were searched.

**Charting methods:**

Data from each article meeting the criteria were pulled into a table, including study design, study population, and setting, aims, methods, outcome measures, and significant findings. A second researcher then reviewed the populated data extraction table.

**Results:**

Overall, 383 papers were found: 7 met inclusion/exclusion criteria. Prevalence rates ranged from 22% to 80% depending on the study cohort. Several conditions were associated with urinary incontinence, including frailty, orthopaedics, stroke, palliative care, neurology, and cardiology. There was a potential positive association between mortality and urinary incontinence, although only two papers reviewed reported mortality.

**Conclusion:**

A dearth of literature determined the prevalence, incidence, and mortality rates for older women admitted to hospitals. Limited consensus on associated conditions was found. Further research is needed to fully explore urinary incontinence in older women during hospital admissions, particularly concerning prevalence/incidence and its association with mortality.

## Introduction

Although urinary incontinence (UI) is not a normal part of ageing, up to 40% of older women (⩾55 years of age) report experiencing UI in everyday life;^1^ the issue may lead to a significant reduction in women’s quality of life, psychological health, confidence, sexuality, and societal inclusion.^2^ This occurs to different degrees of severity; some women experience infrequent leakage, and others experience more frequent problems or total inability to control their bladder function.^3^ Despite the known prevalence of UI and its significant impact within community settings, very little is known about UI for women admitted to hospital.

Causes of UI for older women vary from functional causes, such as damage to the urethra, or pelvic floor muscles,^4^ to other issues, including an overactive bladder or lower urinary tract and bladder infections. The likelihood is increased for those who have experienced vaginal birth, are obese, or have familial risk.^5^ Certain medications also increase the risk of UI,^6^ as do chronic clinical conditions, such as arthritis, stroke, Parkinson’s disease, multiple sclerosis, and dementia.^7^

Within the community, some women diagnosed with diabetes, heart failure, hypertension, neurological conditions, and respiratory disease report exacerbation of UI symptoms.^6^ This suggests that women with UI may experience heightened UI symptoms during a hospital admission due to exacerbating existing or new comorbid conditions.

The impact of UI in older women can be significant, with UI reported as a contributing factor to both falls and the development of pressure ulcers.^8^ Likewise, immobility and difficulty accessing toileting facilities in a timely way have been linked to falls.^9,10^ In addition, falls and pressure ulcers are associated with frailty and increased mortality in older adults.^11^ It is therefore not surprising that UI is strongly associated with mortality in the general population^12^ and could be associated with UI in older women during hospital admission.

This scoping review assesses the current evidence base concerning older women’s UI during hospital admissions. Specifically, this review aims to identify the prevalence and incidence of UI, associations between UI and specific health conditions, and associations between UI and mortality.

## Method

The methodological framework developed by Arksey and O’Malley^13^ was used for this scoping review, and the Preferred Reporting Items for Systematic reviews and Meta-Analyses extension for Scoping Review (PRISMA-ScR) were followed^14^ (see supplemental material). The authors did not register the review protocol as this step is not required for scoping reviews.^14^ The overarching aim was to establish what is known from the existing literature about women (⩾55 years of age) experiencing UI during a hospital admission. Specifically, our research questions were as follows:

- What is the prevalence and incidence of UI in older women during hospital admissions?
- What health conditions are associated with UI in older women during hospital admission?
- What mortality rates are directly or indirectly associated with UI in older women during hospital admission?

A search strategy was developed in consultation with an academic librarian. Databases included CINAHL, MEDLINE, and Cochrane and exploded MESH terms were developed, including the identification of ambiguous terms and potential synonyms (see supplemental material). The final search of peer-reviewed published literature was run in September 2021. To capture the most up-to-date data on UI, the search was limited to studies conducted between 2015 and 2021. Only English-language articles were included due to funding and time constraints. Our research aimed to explore UI in older women across all conditions; however, it was decided that papers that included data for males, younger adults, and focussed on specific conditions would be included if older women were included in the sample. Table 1 lists the inclusion and exclusion criteria below.

**Table 1.** Inclusion and exclusion criteria.

Criteria	Include	Exclude
Publication date	• Studies conducted between 2015 and 2021	• Studies conducted prior to 2015
Language	• Articles written in English	• Articles not written in English
Country	• Studies conducted in any country	
Topic	• Studies which assessed incidence and prevalence* of UI, and related mortality and conditions during hospital admission	• Studies which assessed the incidence and prevalence of UI, and related mortality in primary care or community-based studies • Studies focussing on the treatment of UI • Studies focussing on faecal incontinence
Study population	• Studies accessing UI across all/any clinical conditions • Studies assessing UI in women > 55 years of age	• Studies only including men • Studies only including women < 55 years of age
Publication type	• All empirical research studies	• Opinion pieces and theoretical Journal articles

* For this review, we defined prevalence as the number/percentage of cases of a UI at a particular time point and incidence as the rate of new cases of a UI occurring over a particular period.

Details for each paper returned from the search were exported into a spreadsheet using Microsoft excel.

### Study selection and data extraction

A researcher read abstracts and reviewed them against the inclusion/exclusion criteria. Any abstracts which were queried for inclusion were discussed with two other researchers. An additional 10% of the abstracts were selected randomly and reviewed by two other researchers to ensure agreement on inclusion. Once consensus was achieved, full articles were retrieved for inclusion consideration. Papers meeting the full criteria were retained for data extraction.

Details for each paper meeting the criteria mentioned above were exported into a table populated by one researcher with the following information: Title, authors, study design, study population, and setting, aims, methods, outcome measures, and significant findings. A second researcher then reviewed the populated data extraction table.

### Quality assessment

Three researchers assessed and agreed on the quality of the included studies using the ‘quality assessment with diverse studies’ (QATSDD) tool.^15^ The QATSDD tool reports quality in reviews of mixed or multimethod studies. This allowed us to use one quality assessment tool to review all studies. The tool uses 16 questions (2 dedicated to quantitative and 2 to qualitative studies). Each question scores 0–3 (3 being higher quality) points, with scores of 0–42 for single methods and 0–48 for mixed methods studies. Percentage scores were also recorded to allow comparison across study types, where a higher percentage equates to better quality. As relatively few studies met our eligibility criteria, the decision was made to include all papers in data extraction, irrespective of quality. Despite this, quality assessment of the studies has still been reported as we wished to provide a quality assessment description to inform future researchers on the quality of literature.

### Data analysis

Due to the heterogeneity of our inclusion criteria and papers included in the review, pooled analysis was not deemed appropriate. As a consequence, we summarized the results of the papers using a narrative descriptive synthesis approach.^13,16,17^

## Results

**Figure 1.**
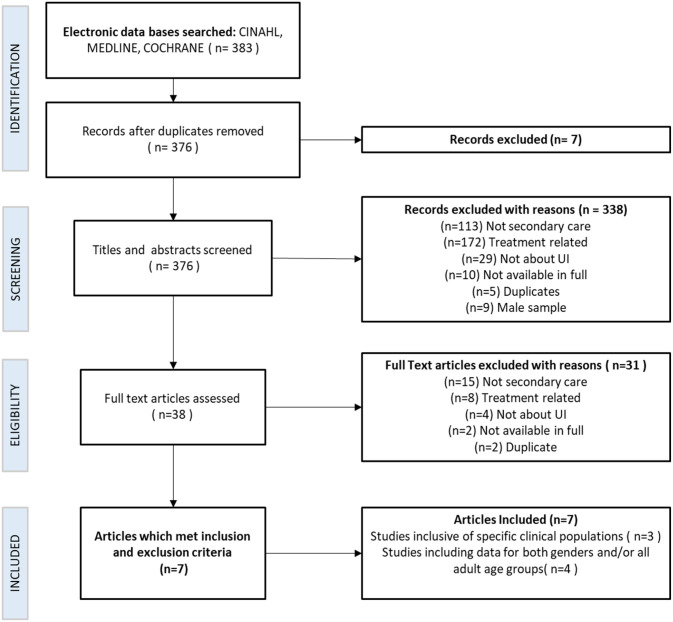
PRISMA flowchart of the selection of included studies in the review.

CINAHL and MEDLINE databases were searched simultaneously, returning 383 papers. The Cochrane database was searched separately and returned no papers. Initially, seven duplicate papers were identified and excluded. All abstracts were then screened, and a further 338 were excluded. Therefore, 38 papers were read in full, and a further 31 papers were excluded. Details, including reasons for exclusion at each stage, are shown in Figure 1. Seven papers met the criteria and were included within two themes: (1) studies inclusive of specific clinical populations and (2) studies including data for both genders and all adult age groups.

### Quality assessment

Total quality scores ranged from 16 to 28 (38.10%–66.67%), demonstrating that overall papers were low to medium quality (see Table 2).

**Table 2.** Overview of study characteristics and quality of papers included.

First author and publication year	Country of study	Study type	UI definition used	QATSDD score
Aly et al.^18^	Egypt	Cross-sectional	Arabic version of ICIQ-UI SF^19^	20 (47.62%)
Truszczyńska-Baszak et al.^20^	Poland	Cross-sectional	ICIQ-UI-SF^19^ ICIQ-LUTSqol.^21^	17 (40.48%)
Long et al.^22^	The United Kingdom	Audit	Incontinence screening questionnaire	16 (38.10%)
Mallinson et al.^23^	The United States	Retrospective cohort	IRF-PAI^24^ – function modifier item for bladder frequency of accidents	27 (64.29%)
Chong et al.^25^	Singapore	Prospective cohort	Patients’ electronic medical records	28 (66.67%)
Condon et al.^26^	Ireland	Cross-sectional observational	Modified Barthel Index^27^	26 (61.90%)
Barakat-Johnson et al.^28^	Australia	Quasi-experimental mixed methods	Clinical assessment	27 (56.25%)

### Studies reporting data for specific clinical populations

Three papers reported on UI in women during hospital admission but focussed on specific patient populations. A description and summary of these papers in relation to our research questions can be found in Table 3, including the strength and significance of any reported relationships. None of these papers reported information on mortality.

**Table 3.** Summary of papers reporting the prevalence of UI within specific clinical populations (n = 3).

First author and publication year	Sample size	Clinical population studied	Mean age	Study aim	Summary of findings in relation to the research questions	
					Prevalence and incidence of UI	Health conditions and health-related factors associated with UI
Aly et al.^18^	130	Frail female patients above 60 years of age	70.7 (SD 8.3)	Investigated the relationship between frailty and UI in women above 60 years of age	80% experienced UI	Relationship between frailty and UI (χ^2^ = 15.511, p < 0.001) and severity of UI (χ^2^ = 50.763, p < 0.001). Participants with UI were older (τ = 4.966, p < 0.001) and had a higher degree of functional impairment (χ^2^ = 8.371, p = 0.015). Relationships between osteoarthritis, stroke, and vaginal prolapse and UI were claimed, but the strength and significance were not reported.
Truszczyńska-Baszak et al.^20^	80	Female patients with LSS	55.1 (SD 12.5)	Assessed the prevalence of UI in female patients with LSS	56% LSS and 43% of clinical control experienced UI	Total ICIQ-UI-SF score: LSS = 4.8 ± 1.4 versus Controls = 2.9 ± 0.8 (p = 0.089). Correlations observed between: ICIQ-UI-SF and ICIQ-LUTSqol (τ = 0.719; p < 0.001) and number of vaginal childbirths and ICIQ-UI-SF (τ = 0.271; p = 0.010)
Long et al.^22^	108	Female psychiatric patients	35.6 (SD 15.6)	Audit of the nature, incidence, and management of incontinence in a secure psychiatric facility for women	45% experienced UI 2% experienced both UI and FI	Stress, urge, and nocturnal UI associated with: smoking (nocturnal UI χ^2^ = 14.22, p < 0.01; stress, χ^2^ = 13.73, p < 0.05; urge, χ^2^ = 12.06, p < 0.05), childbirth (nocturnal UI, χ^2^ = 13.11, p < 0.01; stress, χ^2^ = 12.73, p < 0.01; urge, χ^2^ = 11.13, p < 0.05), and clozapine (nocturnal: χ^2^ = 13.7, p < 0.01, stress: χ^2^ = 14.27, p < 0.01, and urge: χ^2^ = 12.76, p < 0.01). Urge and nocturnal UI associated with obesity (Urge: χ^2^ = 11.73, p < 0.05; nocturnal UI: χ^2^ = 11.18, p < 0.01). Nocturnal UI associated with: diabetes (χ^2^ = 13.11, p < 0.01), epilepsy (χ^2^ = 13.73, p < 0.01), cardiovascular accident (χ^2^ = 14.03, p < 0.01), asthma (χ^2^ = 13.72, p < 0.01), hypertension (χ^2^ = 13.01, p < 0.01), neurological conditions (χ^2^ = 12.75, p < 0.01), and dementia or cognitive impairment (χ^2^ = 13.14, p < 0.01)

Aly et al.^18^ investigated the relationship between frailty and UI in women above the age of 60 years. The prevalence of UI in studied patients was 80%. They found a significant relationship between UI and frailty, and between the severity of frailty and the severity of UI. Participants with UI were significantly older and had a higher degree of functional impairment. The authors reported that quality of life was pointedly impaired, particularly for those experiencing mixed-type UI and longer duration of symptoms of UI. UI was also associated with osteoarthritis, stroke, and vaginal prolapse, although information on the strength and significance of this relationship was not reported.

Truszczyńska-Baszak et al.^20^ assessed the prevalence of UI in female patients with lumbar spinal canal stenosis (LSS). Overall, 56% of women with LSS experienced UI compared to 43% of the clinical control group. A difference approaching significance was reported for total points scored on the ICIQ-UI-SF^19^ between the LSS and control groups. Quality of life was measured with the ICIQ-LUTSqol,^21^ and a significant correlation was observed between the total score of the ICIQ-UI-SF^19^ and the total score of the ICIQ-LUTSqol.^21^ A significant correlation was also reported between the number of vaginal childbirths and the total score of ICIQ-UI-SF.^19^

Long et al.^22^ conducted a study in a female secure psychiatric hospital to examine the rate and management of incontinence and its relationship with psychopathology. However, 45% of patients experienced UI, 2% experienced both UI and faecal incontinence (FI), and 1% suffered from FI only. Meanwhile, 24% experienced urge incontinence, 40% experienced stress incontinence, and 22% experienced both stress and urge incontinence. Associations between physical attributes/health conditions and three types of UI – stress, urge, and nocturnal – were explored. All three were shown to be significantly associated with smoking and childbirth. They also revealed that obese patients were likelier to suffer from nocturnal and urge UI. Nocturnal UI was also associated with several health conditions, including diabetes, epilepsy, cardiovascular accident, asthma, hypertension, neurological conditions, and dementia/cognitive impairment. Clozapine was associated with all three types of UI. Authors also reported that incontinence pads were used in 27% of all patients; five no longer had an incontinence problem, and six reported a ‘minor’ problem that resulted in pad use as a preventive measure. Alternative management methods included medication (21%) and pelvic floor/core stability exercises (13%). Referrals to a hospital consultant occurred in 46% of UI cases.

### Studies reporting data for both genders and all adult age groups

Four studies reported prevalence rates for UI in patients across clinical conditions but for both genders and all adult age groups. A description and summary of these papers in relation to our research questions can be found in Table 4, including the strength and significance of any reported relationships.

**Table 4.** Summary of papers reporting data for both genders and all adult age groups (n = 4).

First author and publication year	Sample size	% Women	Mean age	Study aim	Summary of findings in relation to the research questions		
					Mortality rates directly or indirectly associated with UI	Prevalence and incidence	Health conditions and health-related factors associated with UI
Mallinson et al.^23^	403,697	63	76.3 (SD 10.2)	Assessed the prevalence of UI in patients discharged from rehabilitation facilities	Non-significant 6-month mortality rate difference of 4% for stroke and orthopaedic patients with UI compared to continent patients.	23.8% of all patients (22.2% of women) were incontinent 43.5% were continent with a device	The most common reasons for admission in the UI cohort were orthopaedic (46%) and stroke (26%). The incontinent group was significantly older than the continent group (p < 0.001). Factors significantly (p ⩽ 0.05) related to remaining incontinent for women were being above 80 years of age, being black, having comorbid conditions, having difficulty with cognition, and having longer rehabilitation length of stay. Models explained 4%–8% of the variance.
Chong et al.^25^	210	69.5	89.4 (SD 4.6)	Evaluated the impact of frailty on new-onset UI among hospitalized older adults.	Underlying UI increased mortality risk (at 6 months: 32.0% versus 9.1%, p < 0.001; and at 12 months: 47.0% versus 19.8%, p < 0.001). Premorbid UI independently predicted mortality (6 months: OR = 3.10, 95% CI = 1.34–7.17, p = 0.008; 12 months: OR 3.41, 95% CI = 1.59–7.32, p = 0.002).	64.8% of frail patients and 30.5% of non-frail patients had UI. Incident UI (frail versus non-frail) at initial hospitalization was 29.7% versus 12.3% (p = 0.025), and at 12-month follow-up, 56.8% versus 33.3% (p = 0.02)	Frail patients were more likely to have a history of UI compared to non-frail patients (64.8% versus 30.5%, p < 0.001). Frailty was an independent predictor of incidence (at discharge: OR 2.98, 95% CI = 1.00–8.91, p = 0.05; at 6 months: OR 2.86, 95% CI 1.13–7.24, p = 0.027; and at 12 months: OR 2.67, 95% CI = 1.13–6.27, p = 0.025).
Condon et al.^26^	435	47	72 (SD 23)	Evaluated the prevalence of UI and FI, and their predictors among inpatients in an acute university hospital on a single day.	No information on mortality included	The prevalence of UI was 26%. Separate prevalence data for women was not reported.	Orthopaedic wards had the highest prevalence (52.2%). Frailty was significantly associated with UI. Patients with UI were significantly older (Median = 76 versus 69, p < 0.001) Patients with UI had higher frailty scores (p < 0.001). Frailty status was an independent predictor of UI (p = 0.0106).
Barakat-Johnson et al.^28^	259	48.4	73.2 (SD 16.8)	Evaluated the impact of prevention initiatives on incontinence-associated dermatitis (IAD) (including the use of barrier cream cloths), incontinence practices, and prevalence	No information on mortality included	47.2% of the sample were incontinent, and the prevalence of UI in women was 50%. The proportion of patients with UI pre versus post (52.3% versus 45.5%) intervention did not change significantly (p = 0.30).	Aged care acute/subacute, rehabilitation and neurology units had the highest rates of patients with UI.

Mallinson et al.^23^ investigated the prevalence of UI within inpatient medical rehabilitation facilities in the United States. These included stroke, brain injury, spinal cord injury, and orthopaedic patients. Approximately 67% of all patients were either incontinent (24%) or continent with a device (44%) (urinary catheter or male urinary sheath) at admission. However, 22% of women were classed as incontinent at admission. The incontinent group was significantly older than the continent group. The most common reasons for admission in the UI cohort were orthopaedic (46%) and stroke (26%). Regression models separated by gender and impairment group were also performed to examine change in continence status. Factors significantly related to remaining incontinent compared to improved continence status for women were being above 80 years of age, being black, having comorbid conditions, having difficulty with cognition, and having longer rehabilitation length of stay. The models were statistically significant; however, they only explained 4%–8% of the variance in change in continence status. An increased 6-month mortality rate of 4% for stroke and orthopaedic patients with UI compared to continent patients was observed; however, this was not statistically significant.

Chong et al.^25^ conducted a study to evaluate the impact of frailty on new-onset UI among hospitalized older adults. They found frail patients were more likely to have a UI history than non-frail patients (65% versus 31%). They also observed that incident UI among previously continent-frail individuals was significantly higher and continued to increase from initial hospitalization (30% versus 12%) to 12-month follow-up (57% versus 33%) compared to non-frail patients. Multiple logistic regression, adjusted for age, sex, and severity of illness, showed that frailty was an independent predictor of incidence of UI at discharge, at 6 and 12 months. Underlying UI also significantly increased mortality risk at 6 and 12 months. Multiple logistic regression, adjusted for age, sex, and severity of illness, revealed that premorbid UI independently predicted mortality at 6 and 12 months.

Condon et al.^26^ conducted a study to evaluate the prevalence of UI and FI, and their predictors among hospital inpatients on a single day. The prevalence of UI was 26%; however, separate prevalence data for women were not reported. Orthopaedic wards had the highest prevalence of patients with UI (52%). Patients with UI were shown to be significantly older (median = 76 versus 69) and had higher frailty scores than those without UI. Logistic regression showed that frailty status was an independent predictor of UI. They reported that only 2% of patients had received comprehensive continence assessments.

Barakat-Johnson et al.^28^ examined the effects of an evidenced-based intervention to reduce incontinence-associated dermatitis (IAD) across four hospitals and 12 wards in a district of Australia. Wards included aged care acute/subacute, palliative care, rehabilitation, neurology, and cardiology. The data showed a UI prevalence of 47% across all patients and 50% in females. Prevalence data separated by age were not available. The proportion of patients with UI pre- (52%) and post (46%)-intervention did not change significantly. Aged care acute/subacute, rehabilitation and neurology units had the highest rates of patients with UI. Focus groups were also conducted with 31 nurses to evaluate using barrier cream cloths as part of the intervention. Themes included benefits to the patient, usability, problems encountered, and related factors, including the importance of being educated on the appropriate use of the cloths and how to identify IAD.

## Discussion

This scoping review was conducted to appraise current UI knowledge for women aged 55 years and older during hospital admissions. Specifically, we wanted to investigate the prevalence and incidence of UI, and the associations between women’s UI, mortality rates, and health conditions. While no individual research paper fully addressed all of our research questions, seven studies gave partial insight into the prevalence/incidence, and associated mortality rates and health conditions of UI for this population.

Across all seven studies included in the review, prevalence rates of UI ranged between 22% and 80%, depending on the study sample. Three studies focussed on specific clinical samples, and four took a broader look at UI across multiple clinical populations/wards in both male and female patients.

Of the studies focussing on specific clinical samples, one study assessing frail female patients above 60 years of age had the most representative cohort for this review and observed the highest prevalence rate at 80%.^18^ This percentage is much higher than the observed prevalence rates of UI in women in community settings,^1^ suggesting the issue of UI in older women during hospital admissions is substantial. However, given that the study sample included exclusively frail women, the confounding factors of frailty and increased age possibly produced an elevated prevalence rate. Other clinically specific samples of female patients observed lower prevalence rates; 56% in patients with lumbar spinal canal stenosis^20^ and 48% in psychiatric patients.^22^ However, both prevalence rates are still higher than those observed in women from community settings, around 40%.^1^ Patients in these studies were less representative of this review’s target sample, particularly the psychiatric cohort, who were significantly younger (M_age_ = 35.6 years, SD = 15.6), and were inpatients within a mental health facility rather than within a hospital.^22^

Of the studies that took a broader look at UI across multiple clinical populations in male and female patients, two observed comparable prevalence rates to those examining specific conditions and two observed much lower prevalence rates. Studies which observed comparable prevalence included a study evaluating the effects of IAD prevention initiatives, which observed a prevalence of 47% across all patients and 50% for women,^28^ and a study comparing frail and non-frail hospitalized adults, which found that frail patients had a prevalence of 65%. In comparison, non-frail patients had a prevalence of 31% (separate prevalence data for women were not reported in this study).^25^ While prevalence rates vary slightly across these studies, all suggest higher rates during hospital admissions compared to community settings.^1^

Conversely, two papers observed much lower prevalence rates; one found that 24% of all patients and 22% of female patients within US rehabilitation facilities were incontinent of urine at admission,^23^ and another reported a 26% prevalence rate across all inpatients (separate data for women were not reported in this study).^26^ One explanation for these lower prevalence rates is the definition used for UI. While UI assessment across studies varied, several studies^18,20,22^ used the ICIQ-UI SF^19^ or an adapted version. This patient-reported outcome measure assesses the frequency and quantity of urine leakage, information on stress and urge, and its impact on the patient’s life. Both studies, which reported lower prevalence rates,^23,26^ used clinician-reported outcome measures, neither of which considered stress versus urge incontinence or the quantity of urine leakage. Therefore, these measures and the clinicians using them may underestimate UI, particularly in those who experience a smaller amount of leakage. This discrepancy is an important finding that requires further research. It also highlights a limitation of this review, as there is no internationally recognized standardized measure of UI or its severity, making it difficult to compare prevalence data for the condition with any accuracy. Another factor which may explain differing prevalence rates is catheterization. Despite having a low prevalence of UI, one study reported that 44% of all patients were categorized as continent with a device (urinary catheter or male urinary sheath) on admission.^23^ This finding highlights a difficulty in gaining true prevalence data on UI during hospital admissions as information on why the catheter was used and what would occur after removal is not always available, potentially leading to underestimation in UI prevalence.

Only two papers discussed mortality rates associated with UI. One reported a non-significant difference (4%) in mortality rates between continent and incontinent stroke, and orthopaedic patients.^23^ The other demonstrated that underlying UI significantly increased mortality risk at 6 and 12 months post-discharge and that premorbid UI independently predicted mortality.^25^ These findings suggest an association between UI and mortality. However, more research is needed to explore this association fully. Both studies included both male and female patients in their cohort. As women are more likely to experience UI,^29^ more focussed research exploring these associations in this group is necessary.

This review also explored which health conditions were associated with UI. Our findings suggest some consensus on which types of patients have the highest prevalence of UI. Most studies suggest orthopaedic, stroke, palliative care, neurology, and cardiology patients have the highest prevalence rates.^18,22,23,26,28^ This is an important finding as it can inform future research and interventions on which wards/clinical specialities should be targeted. Frailty also significantly impacted UI in several studies regarding prevalence, incidence, and as a predictor of patient outcomes.^18,25,26^ One study that specifically compared UI in frail and non-frail hospitalized patients found that frailty independently predicted the incidence of UI, which aligns with previously described findings, showing elevated prevalence rates in frail individuals.^18^ Frailty should therefore be considered in future UI research, mainly as a potentially increased mortality rate is associated with frailty and UI.

Overall, this review revealed that relatively few studies had been conducted to explore the nature and extent of the UI issue during hospital admissions and even fewer focussed purely on older women. Although both men and women experience UI, the physiology, aetiology, and clinical reasons differ. It is therefore important to undertake dedicated research for each of the sexes to better understand the prevalence, mortality, and clinical conditions most likely associated with UI, so that suitable interventions for each can be addressed. While seven papers were included in our review, no individual research papers fully addressed all our research questions. Regarding prevalence and incidence, rates varied depending on the cohort studied, and the assessment and classification system used to define UI. Future research should explore these differences and why clinician-completed assessments may lead to underreporting of UI prevalence. Some consensus was reached across studies regarding associated conditions, particularly frailty, which appears to significantly impact UI. Patient groups with the highest prevalence of UI include orthopaedic, stroke, palliative care, neurology, and cardiology, illustrating that future research and UI interventions may be best targeted and most effective in wards of these specialities. Mortality rates were only reported in two papers reviewed but suggested a potential association between UI and mortality rates. Further research is needed to explore the nature of this relationship fully.

### Strengths and limitations

To our knowledge, this is the first review to assess knowledge of UI in older women during hospital admission. This review has highlighted a significant gap in knowledge regarding the prevalence of UI within this population and a lack of evidence regarding the association between UI incidence and specific conditions, mortality and morbidities for these women. A scoping review methodology was chosen to give an expansive view of a complex issue. However, the heterogeneous nature of the data collected prevents meta-analysis, and the research question would need to be narrowed down for a systematic review to be possible. As there is no internationally recognized, standardized measure of UI or its severity, comparing prevalence data for the condition between studies with any accuracy is difficult. The studies described in this review are also from different countries with different healthcare systems and infrastructure which may not be entirely generalizable to other countries.

## Conclusion

A dearth of literature explored UI in older women during hospital admissions. Most studies in this review suggest a higher prevalence of UI during hospital admissions than in community settings, although rates varied depending on the cohort studied. Targeted research assessing UI prevalence during hospital admissions for this demographic across all clinical conditions is needed. Results of this review suggest that using clinician-completed assessments of UI could lead to underreporting of UI prevalence, which should be considered and explored in future research. In terms of comorbidities and mortality associated with UI, frailty appears to significantly impact UI prevalence during hospital admissions, and a potential association between UI and mortality rates has been observed. However, further research is needed to fully explore this relationship’s nature.

## Supplemental Material

sj-docx-1-whe-10.1177_17455057231179061 – Supplemental material for Urinary incontinence in women 55 years and older: A scoping review to understand prevalence, incidence, and mortality of urinary incontinence during secondary care admission Click here for additional data file. Supplemental material, sj-docx-1-whe-10.1177_17455057231179061 for Urinary incontinence in women 55 years and older: A scoping review to understand prevalence, incidence, and mortality of urinary incontinence during secondary care admission by Isobel McMillan, Lyndsay Hill, Robyn McCarthy, Ruth Haas-Eckersley, Margaret Russell, Julie Wood, Liz Doxford-Hook, Yu Fu, Linda McGowan and Heather Iles-Smith in Women’s Health

## Declarations

**Ethical approval and consent to participate:** Not applicable.

**Consent for publication:** Not applicable.

**Availability of data and material:** Not applicable.
