# Supplementary material for: Urinary incontinence in women 55 years and older: A scoping review to understand prevalence, incidence, and mortality of urinary incontinence during secondary care admission
Source: Womens Health (Lond). 2023 Jun 16;19:17455057231179061. doi: 10.1177/17455057231179061 (PMC10278415; doi:10.1177/17455057231179061)
Supplement: sj-docx-1-whe-10.1177_17455057231179061 – Supplemental material for Urinary incontinence in women 55 years and older: A scoping review to understand prevalence, incidence, and mortality of urinary incontinence during secondary care admission [file sj-docx-1-whe-10.1177_17455057231179061.docx]

# Supplementary material

Table 1. Exploded MESH terms used for database searches

|  |  | Number of results |
| --- | --- | --- |
|  | CINAHL |  |
|  | AB Fatalit* OR AB fall* OR AB death* OR AB incidence OR AB prevalence OR AB pressure sore* OR AB mortalit* |  |
|  | AB In-patient OR AB Hospital stay OR AB Tertiary care OR AB secondary care OR AB on ward |  |
|  | AB wom* OR AB female* |  |
|  | AB urinary incontinence OR AB stress incontinence OR AB urge incontinence OR AB urinary continence OR AB urinary voiding |  |
|  |  |  |
|  | Exploded |  |
| 1 | (MH "Urinary Incontinence+") OR (MH "Urge Urinary Incontinence (Saba CCC)") OR (MH "Total Urinary Incontinence (Saba CCC)") OR (MH "Stress Urinary Incontinence (Saba CCC)") OR (MH "Reflex Urinary Incontinence (Saba CCC)") OR (MH "Functional Urinary Incontinence (Saba CCC)") OR (MH "Incontinence Aids+") OR (MH "Stress Incontinence") OR (MH "Reflex Incontinence (NANDA)") | (12,478) |
|  |  |  |
| 2 | (MM "Fatal Outcome") OR "fatalities"OR (MH "Death+") OR "death" OR (MH "Prevalence") OR "prevalence" OR (MH "Epidemiology+") OR (MH "Accidental Falls") OR "fall*" OR (MH "Pressure Ulcer") OR "pressure sore" OR (MH "Incidence") OR "incidence" OR (MH "Mortality") OR (MM "Hospital Mortality") | (1,588,213) |
|  |  |  |
| 3 | (MM "Women") OR (MH "Female") OR "female*" | (2,129,671) |
|  |  |  |
| 4 | (MH "Inpatients") OR (MH "Hospitalization+") OR "hospital stay" OR (MH "Secondary Health Care") OR "secondary care" OR (MH "Nursing Care Plans") OR "on ward" OR “tertiary care” | (2,353,415) |
|  |  |  |
|  | 1 AND 2 AND 3 AND 4 | (1,372) |
|  |  |  |
|  | Date 2015-2021 | 450 |
|  |  |  |
|  | Age 45+ and English language | 176 |
|  |  |  |
|  | ADD Medline Data base | 383 |
|  |  |  |
|  |  |  |
|  | CINAHL +MEDLINE Exclusions |  |
|  |  |  |
|  |  |  |

|  |  |  |
| --- | --- | --- |
| Cochrane | “Urinary Incontinence” AND (WOm* OR Female*) AND (“secondary care” OR “Admission*”) AND (Prevalence OR Incidence)  (2015-2021) | 0 |
|  | “Urinary Incontinence” AND (WOm* OR Female*) AND (“secondary care” OR “Admission*”) AND (Prevalence OR Incidence)  (2015-2021) | 0 |
